# Olmesartan Attenuates Single-Lung Ventilation Induced Lung Injury *via* Regulating Pulmonary Microbiota

**DOI:** 10.3389/fphar.2022.822615

**Published:** 2022-03-23

**Authors:** Di Lu, Zhizhi Wang, Zhiming Chen, Jiayang Fan, Jianxue Zhai, Duopei Fang, He Cai, Xiguang Liu, Hua Wu, Kaican Cai

**Affiliations:** Department of Thoracic Surgery, Nanfang Hospital, Southern Medical University, Guangzhou, China

**Keywords:** pulmonary microbiota, metabolite, single-lung ventilation, lung injury, angiotensin receptor blocker, olmesartan

## Abstract

Single-lung ventilation (SLV) associated acute lung injury is similar to ischemia reperfusion (IR) injury which is usually occurred during lung surgery. Olmesartan (Olm), a novel angiotensin receptor blocker (ARB), has been reported to ameliorate organ IR injury. Several recent studies have shown that lung microbiota may be involved in pulmonary diseases, but the effect of pulmonary microbiota in SLV-induced lung injury has not been reported. This study aims to determine the mechanism of how Olm attenuates SLV induced lung injury. Our data showed that 7 days Olm treatment before modeling markedly alleviated SLV-induced lung injury by suppressing inflammation and reactive oxygen species. Bronchoalveolar lavage fluid samples from the injured side were collected for 16S rRNA gene-based sequencing analysis and 53 different bacteria at the genus and species levels were identified. Furthermore, the injured lung samples were collected for metabolomics analysis using liquid chromatography-mass spectrometry analyses to explore differential metabolites. The Kyoto Encyclopedia of Genes and Genomes (KEGG) was applied to analyze the correlation between differential metabolites and lung microbiota. A total of 38 pathways were identified according to differential metabolites and 275 relevant pathways were enriched via analyzing the microbial community, 24 pathways were both identified by analyzing either metabolites or microbiota, including pyrimidine metabolism, purine metabolism, aminoacyl-tRNA biosynthesis and ATP-binding cassette transporter. Besides classical blockage of the renin-angiotensin II system, Olm could also alleviate SLV-induced lung injury by rewiring the interaction between pulmonary microbiota and metabolites.

## Introduction

Single-lung ventilation (SLV) is a widely used technique whereby one lung is excluded from ventilation while perfusion to the non-ventilated lung is continued (Bignami et al., 2018). It is a unique technique in thoracic surgery as it is favored by the increasing use of minimally invasive techniques (Lohser and Slinger, 2015). However, SLV is believed to induce acute lung injury, as it has been reported that SLV is associated with 20% postoperative pulmonary complications (Licker et al., 2006). The pathophysiologic mechanisms underlying such complications are multifaceted, such as hypoxic pulmonary vasoconstriction during ischemia, the production of oxidative stress and inflammation after reperfusion, which is similar to the process of ischemia reperfusion (IR) injury (Chiang et al., 2008; Ferrari and Andrade, 2015; Lohser and Slinger, 2015).

Several strategies have been reported to reduce such damage, including the application of the angiotensin receptor blocker (ARB) (Tanaka et al., 2013). ARBs are widely used to treat hypertension and recommended by several guidelines as they are able to decrease blood pressure and protect target organs by blocking the angiotensin II type-1 receptor (Wang et al., 2016; Nerenberg et al., 2018; Whelton et al., 2018; Williams et al., 2018). Olmesartan (Olm) is a novel ARB with a unique structure and efficacy. In addition to its general role as an ARB, Olm has been reported to protect against cardiac damage and renal injury (Dai et al., 2010; Michel et al., 2016). To date, there was no study investigating the protective effect of Olm against lung injury.

The existence of respiratory tract flora was widely accepted recently (Dickson et al., 2016). Researchers have demonstrated that host bacterial colonizers play a key role in maintaining normal metabolism and promoting the development of the host immune system (Fagundes et al., 2012; Man et al., 2017; Yin et al., 2017). For instance, several resident bacteria, such as *Prevotella* and *Oscillibacter*, were able to release their metabolites which contribute to the suppression of inflammation and ameliorate the damage caused by oxidative stress (Iino et al., 2007; Zhu et al., 2010; Trompette et al., 2014; Larsen et al., 2015).

Moreover, recent studies found that microbiota could affect the therapeutic effect of some drugs. It was reported that gut microbiota could interact with indoxyl sulfate in renal tubular cells to induce reactive oxygen species (ROS) in chronic kidney disease (Devlin et al., 2016). Another recent study showed that vaginal bacteria modified the microbicide efficacy of tenofovir against human immunodeficiency virus in African women (Klatt et al., 2017). Furthermore, recent clinical trials have indicated that antibiotic-induced disruption of the microbiota might impact the efficacy of the immune checkpoint inhibitor *atezolizumab* (Chalabi et al., 2020).

However, it is unknown whether Olm can protect the lung against SLV-induced injury, or whether resident respiratory bacterial colonizers are involved in this pathophysiological process. In this study, we aim to investigate the effect of Olm on SLV-induced lung injury and whether the respiratory tract flora is involved in such process.

## Materials and Methods

### Animals and Treatments

24 male Sprague Dawley (SD) rats (7,8 weeks old; 250–350 g) were bought from the Laboratory Animal Center of Southern Medical University. The animals were housed in a standard laboratory environment (temperature, 22 ± 1°C; humidity, 60 ± 10%, light, 12 h/day) and had free access to food and water. All animal experiments were performed according to the protocol approved by the Animal Care Committee of Nanfang Hospital, Southern Medical University of China. The rats were randomly divided into four groups (*n* = 6 in each group): group I (injury), group AI (ARB + injury), group S (Sham) and group AS (ARB + Sham). Groups I and AI were treated with SLV for 1 h and then double lung mechanical ventilation for 3 h. Groups AI and AS were given Olm (10 mg/kg) for 7 days by intragastric administration before mechanical ventilation. Group S did not receive pretreatment. Following pretreatment, thoracotomy was performed under anesthesia. Blood pressure was measured in all rats every 2 days. Fecal samples were collected before surgery and immediately frozen at −80°C until DNA extraction.

SLV was performed according to the method described by Kentaro et al. (Tojo et al., 2016). The rats were first anesthetized (sodium pentobarbital, 50 mg/kg, i.p.) and then a tracheotomy was performed and a rubber cannula with a broadened tip was inserted into the trachea (Dumas et al., 2007; Zhang et al., 2020). The cannula was further inserted into the right main bronchus and SLV was initiated. The settings were as follows: tidal volume 4 ml/kg; ventilation frequency 80/min. Left posterolateral thoracotomy from the fifth intercostal space was performed, so it was advisable to observe the collapse of the left lung and then confirm that the cannula was in the correct position in the right main bronchus (Tekinbas et al., 2007). After that, the cannula was pulled out to a suitable position and double lung ventilation was continued for 3 h. The settings were the same as those mentioned previously except the tidal volume was 8 ml/kg. When total mechanical ventilation was complete, the lungs were exposed after bilateral thoracotomies.

### Serum Analysis

When the surgery was complete, apical blood was obtained using a heparinized syringe. The serum was obtained after centrifugation (3,000 r/min, 10 min). An enzyme-linked immunosorbent assay (ELISA) kit (Cusabio) was used to determine the concentration of interleukin-6 (IL-6), which is an inflammatory cytokine. In addition, serum malondialdehyde (MDA) was also measured using a similar method (Gorog et al., 1991).

### Collection of Bronchoalveolar Lavage Fluid Samples

After ventilation, with the right main bronchus clipped, left lobe BALF samples were collected by instilling 2 ml sterile saline through the cannula. The resulting pellet was obtained after centrifugation (12,000 r/min, 10 min), and immediately frozen at −80°C for further DNA extraction.

### Histological Examination

The left lung of each rat was collected. The lower half of the collected tissue was weighed, shredded with tissue scissors, and immediately snap frozen in liquid nitrogen. The samples were stored at −80°C. A small area of the upper half of the left lung was separated and 4% buffered formaldehyde solution was added to fix the samples. The samples were then paraffin embedded, and cut into slices 3 μm thick. Hematoxylin-eosin (HE) staining was then performed. Histological scoring of lung injury was carried out by pathologists who were blind to the group allocation using the method of the American Association of Thoracic Surgery Lung Injury Pathology Scoring System (Supplementary Table S1) (Matute-Bello et al., 2011). The remaining left lung was used for measurement of the lung wet weight/dry weight (W/D) ratio.

### Cell Culture

Both human umbilical vein endothelial cells (HUVECs) and human adenocarcinoma alveolar basal epithelial cells (A549) were cultured in 1,640 medium (Gibco) with 10% fetal bovine serum (Gibco). All the cells were cultured in a humidified incubator with 5% carbon dioxide at 37°C. Experiments were conducted when the cells reached 80–90% confluence on Petri dishes. Subsequently, serum-free culture medium was used for 12 h to ensure that the cells were in synchronous growth and a quiescent state. In the Olm-treated groups, cells were treated with 10–6 M olmesartan (Shanghai Macklin Biochemical Company) for 24 h and DMSO was added to the cells as a control treatment. Cell culture supernatants were collected and centrifuged for 20 min at 1,000×g. ELISA was used to determine the concentration of interleukin-1β(IL-1β), IL-6 and tumor necrosis factor-a (TNF-α).

### Hypoxia-Reoxygenation/Ischemia Reperfusion Model

The IR model was employed as the SLV model *in vitro*. Cultures were incubated in a low oxygen environment (5% CO^2^, 5% O^2^, 90% N^2^) for first 1 h. After 1 h anoxia, 3 h reoxygenation treatment was carried out in a 37°Ccell incubator with 21% oxygen and 5% carbon dioxide^30^.

### Quantitation of Cellular Reactive Oxygen Species Level

The ROS level in cells was measured by quantitating the oxidative conversion of cell permeable 2′,7′-dichlorofluorescin diacetate (DCFH-DA) (Sigma Chemicals) to fluorescent dichlorofluorescein (DCF), which was described in a previous report 31. DCFH-DA was prepared by dilution in DMSO at 1:1,000 in advance. When the treatment was complete, HUVECs/A549 were washed, trypsinzed and incubated with 10 μmol/L DCFH-DA in a light-protected humidified chamber at 37°C for 30 min. The cells were then washed twice with PBS and then analyzed by a BD FACSVerseTM flow cytometer (Becton-Dickson, Franklin Lakes, NJ, United States). The excitation wavelength for DCF was 470 nm with emission at 530 nm.

### Metabolomics Analysis of the Lung Samples

Lung samples (50 mg) were weighed and placed in Eppendorf tubes. Following the addition of 1,000 μl of extract solvent (acetonitrile-methanol-water, 2:2:1, containing internal standard), the samples were vortexed for 30 s, homogenized at 45 Hz for 4 min, and sonicated for 5 min in an ice-water bath. Homogenization and sonication were repeated 3 times, followed by incubation at −20°C for 1 h and centrifugation at 12,000 rpm and 4°C for 15 min. The resulting supernatants were transferred to liquid chromatography-mass spectrometry (LC-MS) vials and stored at −80°C until UHPLC-QE Orbitrap/MS analysis. The quality control (QC) sample was prepared by mixing an equal aliquot of the supernatants from all the samples.

LC-MS/MS analyses were performed using an UHPLC system (1,290, Agilent Technologies) with a UPLC HSS T3 column (2.1 mm × 100 mm, 1.8 μm) coupled to a Q Exactive mass spectrometer (Orbitrap MS, Thermo). The mobile phase A was 0.1% formic acid in water for positive mode, and 5 mmol/l ammonium acetate in water for negative mode, and the mobile phase B was acetonitrile. The elution gradient was set as follows: 0 min, 1% B; 1 min, 1% B; 8 min, 99% B; 10 min, 99% B; 10.1 min, 1% B; 12 min, 1% B. The flow rate was 0.5 ml/min. The injection volume was 2 μL. The QE mass spectrometer was used due to its ability to acquire MS/MS spectra on an information-dependent basis (IDA) during an LC/MS experiment. In this mode, the acquisition software (Xcalibur 4.0.27, Thermo) continuously evaluates the full scan survey MS data as it collects and triggers the acquisition of MS/MS spectra depending on preselected criteria. ESI source conditions were set as follows: sheath gas flow rate was 45 Arb, aux gas flow rate was 15 Arb, capillary temperature was 320°C, full MS resolution was 70,000, MS/MS resolution was 17,500, collision energy was 20/40/60 eV in the NCE model, spray voltage was 3.8 kV (positive) or −3.1 kV (negative), respectively.

### 16S ribosomal RNA Gene Sequencing Analysis

The 16 small subunits of ribosomal RNA (16S rRNA) gene are universally present in bacteria and absent in mammals, and gene sequencing was performed to analyze the taxonomic composition of the microbial community in the BALF and fecal samples. Total DNA was extracted using the HiPure Soil DNA Kits (or HiPure Stool DNA Kits) (Magen, Guangzhou, China). The 16S rDNA V3-V4 region of the ribosomal RNA gene was amplified by PCR (95°C for 2 min, followed by 27 cycles at 98°C for 10 s, 62°C for 30 s, and 68°C for 30 s and a final extension at 68°C for 10 min) using primers 341F: CCTACGGGNGGCWGCAG; 806R: GGACTACHVGGGTATCTAAT, where the barcode was an eight-base sequence unique to each sample. Amplicons were extracted from 2% agarose gels and purified using the AxyPrep DNA Gel Extraction Kit (Axygen Biosciences, Union City, CA, United States) according to the manufacturer’s instructions. They were then quantified using an ABI StepOnePlus Real-Time PCR System (Life Technologies, Foster City, United States). Purified amplicons were pooled in equimolar and paired-end sequenced (2 × 250) on an Illumina platform according to standard protocols. Guangzhou Genedenovo Biotechnology Co., Ltd assisted in 16S rRNA sequencing. All raw data was deposited in the SRA of the NCBI (https://www.ncbi.nlm.nih.gov/sra) under accession number SRR14448057 to SRR14448080.

### Statistical Analysis

The raw data of MS analysis were converted to the mzML format using ProteoWizard, and processed by R package XCMS (version 3.2), including retention time alignment, peak detection, and peak matching. The data were then filtered using the following criterion: sample numbers containing a metabolite were less than 50% of all sample numbers in a group (QC was also taken as a group). OSI-SMMS (version 1.0, Dalian Chem Data Solution Information Technology Co., Ltd.) was employed for peak annotation after data processing with an in-house MS/MS database. The multivariate analysis used included principal component analysis (PCA), partial least squares discriminant analysis (PLS-DA) and orthogonal projection to latent structures-discriminant analysis (OPLS-DA). Furthermore, a variable importance in projection (VIP) score of (OTUs) in the PLS model was applied to rank the metabolites that best distinguished between two groups. Those with a *p*-value from the *t*-test < 0.05 and VIP ≥1 were considered differential metabolites between two groups. The Kyoto Encyclopedia of Genes and Genomes (KEGG) was applied to define the significantly enriched pathways in differential metabolites.

For sequencing analysis data, raw reads were further filtered according to the following rules using FASTP (https://github.com/OpenGene/fastp) to obtain high quality clean reads. The removal protocols included: removing reads containing more than 10% of unknown nucleotides (N); removing reads containing less than 80% of bases with quality (*Q*-value) > 20. Paired-end clean reads were merged as raw tags using FLSAH (version 1.2.11) with a minimum overlap of 10 bp and mismatch error rates of 2%. To obtain the high-quality clean tags, noisy sequences of raw tags were filtered by QIIME (version 1.9.1) pipeline under specific filtering conditions. Clean tags were searched against the reference database (http://drive5.com/uchime/uchime_download.html) to perform reference-based chimera checking using the UCHIME algorithm (http://www.drive5.com/usearch/manual/uchime_algo.html). All chimeric tags were removed and finally obtained effective tags were used for further analysis. The effective tags were clustered into operational taxonomic units (OTUs) of ≥97% similarity according to UPARSE pipeline. The representative sequences were classified into organisms by a naive Bayesian model using RDP classifier (version 2.2) based on the SILVA Database (https://www.arb-silva.de/), with confidence threshold values ranging from 0.8 to 1. Visualization of the biomarkers found on taxonomic trees provides an effective tool for concluding the results in a biologically meaningful manner. Alpha diversity analysis was performed by calculating and comparing Chao1, Simpson and all other alpha diversity indices among groups in QIIME. Beta diversity analysis was also performed to express the response of biological species to environmental heterogeneity. The KEGG pathway analysis of the OTUs was inferred using Tax4Fun (version 1.0).

### Correlation Analysis Between Metabolomic Signatures and Microbial Community Profiling

In order to integrate the microbiota and metabolomic data, we performed Two-way Orthogonal Partial Least Squares (O2PLS) analysis. O2PLS models were constructed using the metabolite abundance dataset and each taxonomy level of the microbiota dataset, including levels of OTU, phylum, order, class, family, genus and species. Microbiota with an abundance lower than 0.1% were filtered. After that, the Pearson correlation coefficient model and canonical correspondence analysis (CCA) were performed in R (version 3.5.1) for further exploration of the relationship between altered microbiota and metabolites.

## Result

### Olmesartan can Attenuate Single-Lung Ventilation Induced Lung Injury

**FIGURE 1:**
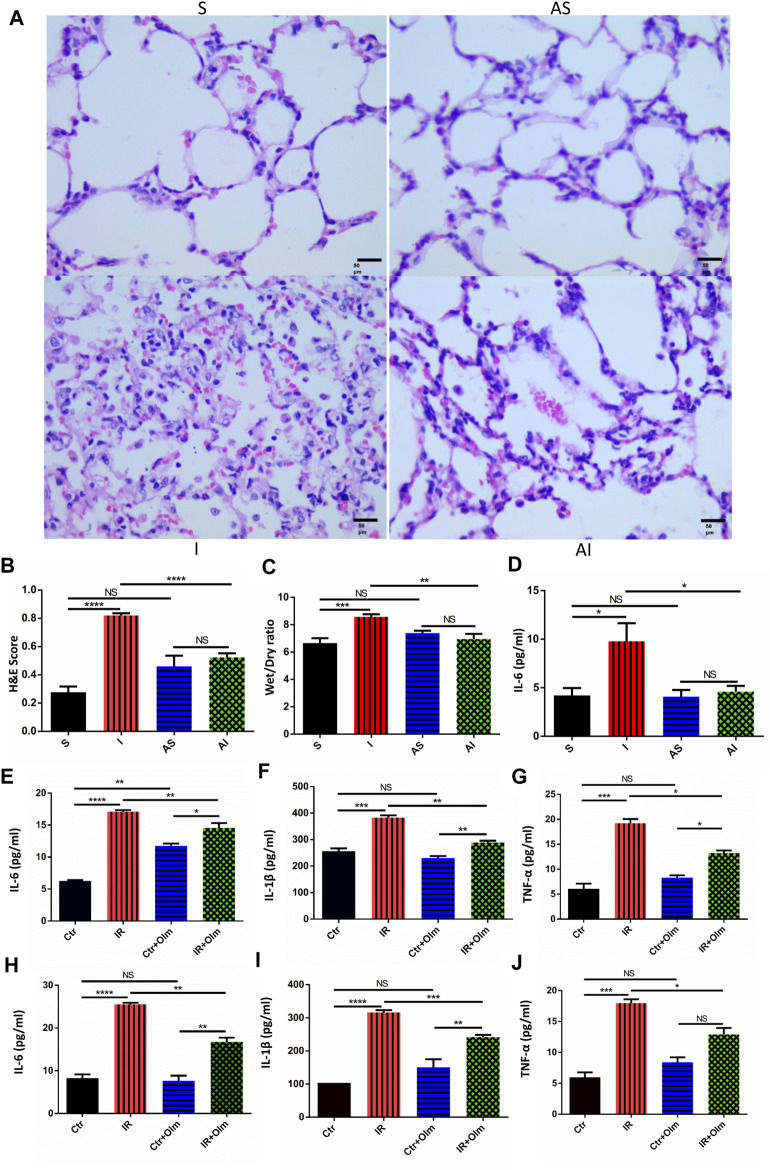
Olmesartan (Olm) can attenuate single-lung ventilation (SLV) induced lung injury. Lung histopathological alterations in rats in the S (sham) group; AS (ARB + sham) group, in which the rats given 7 days Olm treatment before the sham surgery; I (injury) group, in which the rats underwent SLV for 1 h (right lung ventilation and left lung collapsed) and double lungs ventilation for 3 h; and the AI (ARB + injury) group (hematoxylin and eosin staining; original magnification, ×400) (*n* = 6) **(A)**. The lung injury score of HE staining in rats in each group **(B)**. Lung W/D ratio in rats in all groups **(C)**. IL-6 in plasma as shown by ELISA **(D)**. Quantitative analysis of IL-6, IL-1β and TNF-α in HUVECs culture supernatant in different groups by ELISA **(E–G)** (*n* = 3). Quantitative analysis of IL-6, IL-1β and TNF-α in A549 cell culture supernatant by ELISA **(H–J)** (*n* = 3). Data are presented as the mean ± standard error of the mean. NS: not significantly different. *p < 0.05, **p < 0.005, ***p < 0.001, ****p < 0.0001. SLV: single-lung ventilation; Olm: olmesartan; ARB: angiotensin receptor blocker; S: sham; AS: ARB + sham; I: injury; AI: ARB + injury; Ctr: control; IR: ischemia reperfusion; W/D: wet/dry ratio; IL-6: interleukin-6; IL-1β: interleukin-1β; TNF-α: tumor necrosis factor-α; ELISA: Enzyme-linked immunosorbent assay.

As shown in Figure 1A, it was obvious that lung tissues after SLV were more severely damaged and showed more inflammatory cell infiltration than after sham-operation, and such injury was attenuated by Olm when the degree of injury in the AI group was compared with that in the I group. Histological scoring, W/D ratio and IL-6 in serum all represented the degree of lung injury. The histological scoring of lung injury induced by SLV was significantly higher than that in group S (*p* < 0.0001), while following the application of Olm, the lung injury score after SLV significantly decreased (*p* < 0.0001) (Figure 1B). The results of W/D ratio and IL-6, IL-1β and TNF-α in serum also revealed that SLV induced severe inflammation and injury, but Olm was able to significantly down-regulate the degree of injury caused by SLV (Figures 1C,D, Supplementary Figures S1A,B).

In the *in vitro* experiment, an IR model was employed to simulate SLV-induced injury in both HUVECs (Figures 1E–G) and human A549 cells (Figures 1H–J), and related cytokines in the cell supernatant in the IR group, including IL-6, IL-1β and TNF-α, were up-regulated compared to that in the control group (Ctr), respectively. Olm treatment down-regulated the cytokines and increased cell viability after IR, but had no significant influence in the Ctr group. As shown by the *in vivo* and *in vitro* models, SLV can lead to severe lung injury and Olm was able to attenuate the injury.

### Olmesartan Inhibits Single-Lung Ventilation Induced Acute Lung Injury by Suppressing Oxidative Stress

**FIGURE 2:**
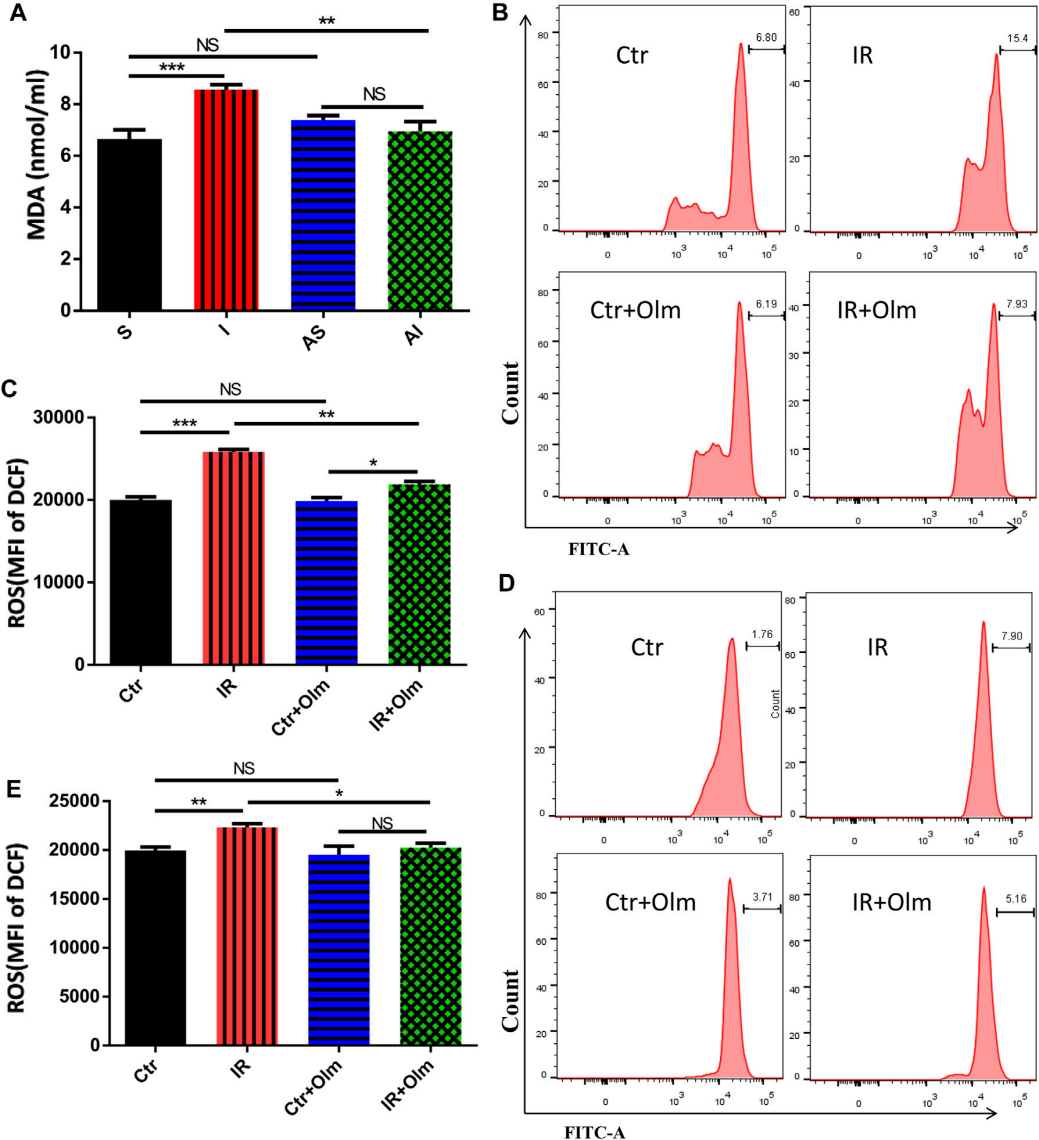
Olmesartan (Olm) inhibits SLV induced acute lung injury by inhibiting oxidative stress. MDA level in serum **(A)** The production of ROS was determined by flow cytometry **(B–D)** (*n* = 3). Representative images of HUVECs **(B)**. Representative images of A549 cells **(C)**. The average MFI of HUVECs **(D)**. The average MFI of A549 cells **(E)**. Data are presented as the mean ± standard error of the mean. NS: not significantly different. *p < 0.05, **p < 0.005, ***p < 0.001, ****p < 0.0001. SLV: single-lung ventilation; Olm: olmesartan; S: sham; AS: ARB + sham; I: injury; AI: ARB + injury; Ctr: control; IR: ischemia reperfusion; MDA: malondialdehyde; MFI: mean fluorescence intensity.

MDA in serum all represented the degree of lung oxidative injury. MDA in serum also revealed that SLV induced severe oxidative damage and injury, but Olm was able to significantly down-regulate the degree of oxidative injury caused by SLV (Figure 2A). The generation of ROS was detected by DCFH assay accompanied by flow cytometric analysis and Dihydroethidium (DHE) staining, and demonstrated similar results in both HUVECs and A549 cells, where ROS were highly up-regulated in the IR group compared with the Ctr group (*p* < 0.005, *p* < 0.05, Figures 2B–E, Supplementary Figures S1C–F). In addition, Olm treatment significantly reduced the generation of ROS in the IR group and did not have a significant effect in the Ctr group or in both HUVECs and A549 cells (Figures 2B–E, Supplementary Figures S1C–F).

### The Composition and Variance of Microbial Communities in all Four Groups

**FIGURE 3:**
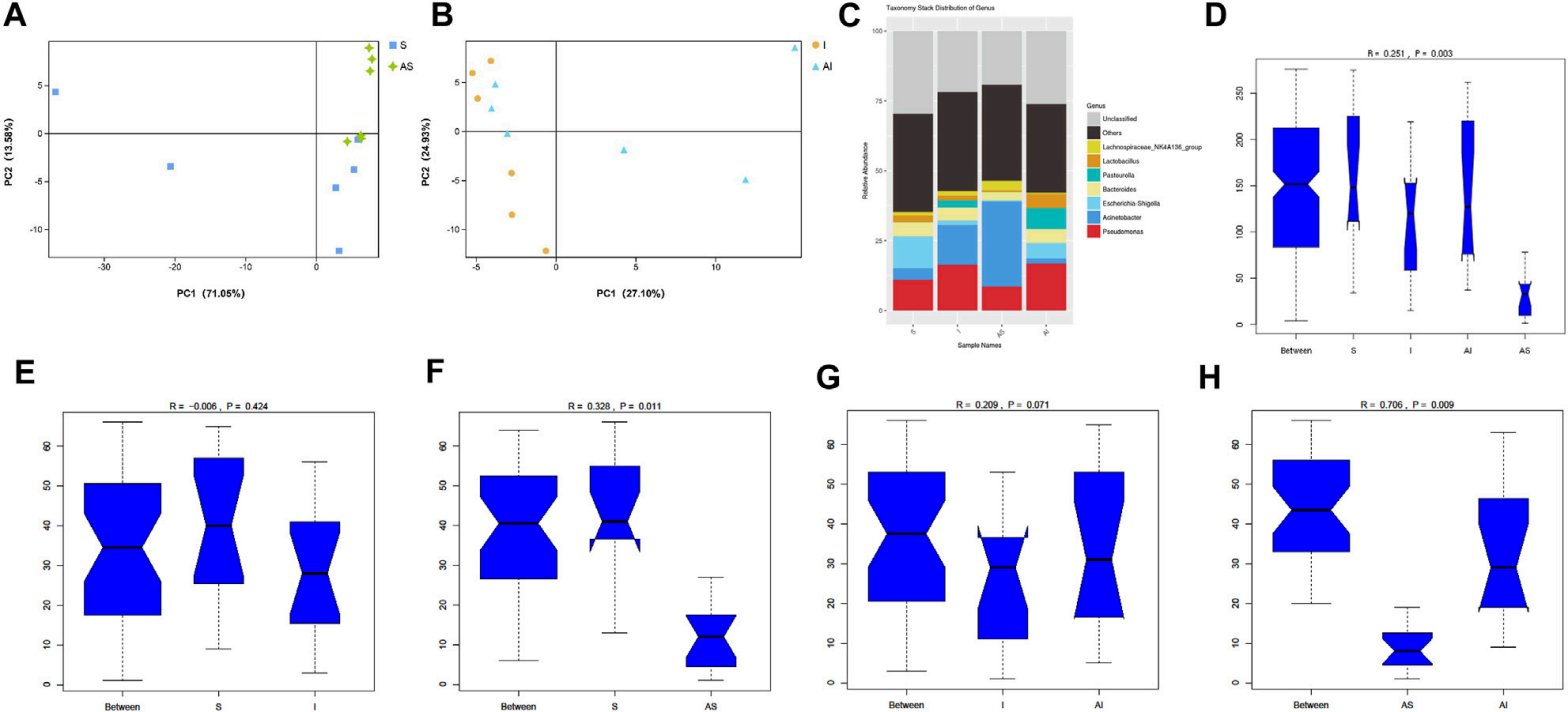
The composition and variance of the microbial communities in all four groups. PCA of bacterial patterns in the lung microbiota community between group S and AS **(A)**, and between group I and group AI **(B)**. The lung microbial community structures of the four groups **(C)**. Boxplots based on the Unweighted Unifrac index were used to show the differences in the mean value of ranks between groups visually **(D–H)**. The analysis was tested using analysis of similarities (Anosim) PCA: principal component analysis; S: Sham; AS: ARB + Sham; I: injury; AI: ARB + injury.

PCA was employed to compare bacterial patterns in the lung and gut microbiota community. The PCA score revealed that the composition of bacterial communities was different between group S and group AS in the lung microbial community, which was also detected between group I and group AI (Figures 3A,B). However, in gut microbiota, no significant difference was found between group AS and group A (see Supplementary Figures S1G–L). The lung microbial community structures in the four groups are shown in Figure 3C and the differences in the distribution between the groups were determined by analysis of similarities (Anosim). Boxplots based on the Unweighted Unifrac index were applied to show the differences in the mean value of ranks between the groups visually (Figures 3D–H). The results showed a significant difference (*p* = 0.003) between all four groups. After further pairwise comparison, it was shown that the differences between group S and group AS, and between group AS and group AI were significant (*p* = 0.011, *p* = 0.009), respectively, while the difference between group S and group I was not significant (*p* = 0.424). Although the analysis showed the difference between group I and group AI was not statistically significant (*p* = 0.071), there was a tendency that the composition and the variance of the microbial communities in group I might be different from that in group AI, as the *p* value was close to 0.05.

### Identification of the Differential Bacteria in the Lung Microbiota

**FIGURE 4:**
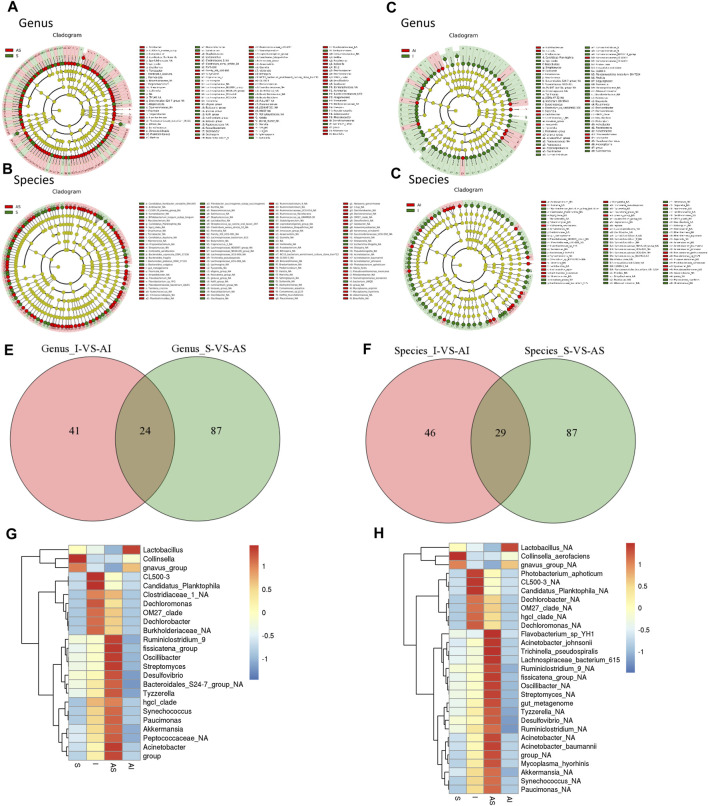
Identification and comparison of the differential bacteria in the lung microbiota from the four groups. The cladograms at both genus and species levels for target differential bacteria between groups S and AS **(A,B)**, and between groups I and AI **(C,D)**. Venn diagrams of shared Operational Taxonomic Units (OTUs) between group I and group AI, and between group S and group AS at both genus and species levels **(E,F)**. Heat maps of both genus and species levels of differential lung microbiota in the different groups **(G,H)**. S: sham; AS: ARB + sham; I: injury; AI: ARB + injury.

To identify the target differential bacteria, it was necessary to evaluate the differential bacteria between the groups. The Kruskal–Wallis rank sum test was performed in all groups, the Wilcoxon rank sum test was used for pairwise comparison, ranking was carried out using Linear Discriminant Analysis (LDA), and then the differences were mapped on a classification tree with a known hierarchy. The final cladograms at both genus and species levels are shown in Figure 4A–D, which revealed the different microbial communities in each group. Figures 4A,B show the difference between group S and group AS, while Figures 4C,D were constructed based on the comparison between group I and group AI (Supplementary Table S2–5). Venn diagrams showed that there were 65 shared OTUs between group I and group AI, 111 shared OTUs between group S and group AS, and 24 shared OTUs between the two comparisons at the genus level (Figure 4E). On the other hand, 29 shared OTUs were identified in the same way (Figure 4F). A total of 53 differences at the genus and species levels were then identified. They were *Acinetobacter, Akkermansia, Bacteroidales_S24-7_group_NA, Burkholderiaceae_NA, Candidatus_Planktophila, CL500-3, Clostridiaceae_1_NA, Collinsella, Dechlorobacter, Dechloromonas, Desulfovibrio, fissicatena_group, gnavus_group, group, hgcI_clade, Lactobacillus, OM27_clade, Oscillibacter, Paucimonas, Peptococcaceae_NA, Ruminiclostridium_9, Streptomyces, Synechococcus, Tyzzerella, Acinetobacter_baumannii, Acinetobacter_johnsonii, Acinetobacter_NA, Akkermansia_NA, Candidatus_Planktophila_NA, CL500-3_NA, Collinsella_aerofaciens, Dechlorobacter_NA, Dechloromonas_NA, Desulfovibrio_NA, fissicatena_group_NA, Flavobacterium_sp_YH1, gnavus_group_NA, group_NA, gut_metagenome, hgcI_clade_NA, Lachnospiraceae_bacterium_615, Lactobacillus_NA, Mycoplasma_hyorhinis, OM27_clade_NA, Oscillibacter_NA, Paucimonas_NA, Photobacterium_aphoticum, Ruminiclostridium_9_NA, Ruminiclostridium_NA, Streptomyces_NA, Synechococcus_NA, Trichinella_pseudospiralis,* and *Tyzzerella_NA*.

The mean relative abundance of the differential lung microbiota in different groups is presented in Figures 4G,H. Except for *gnavus_group, Collinsella and Lactobacillus*, the relative abundance of all bacteria in group I was higher than in group AI. After Olm treatment, the relative abundance returned to normal, indicating that Olm could alleviate the changes in these bacteria.

### Metabolic Variations of Different Groups

**FIGURE 5:**
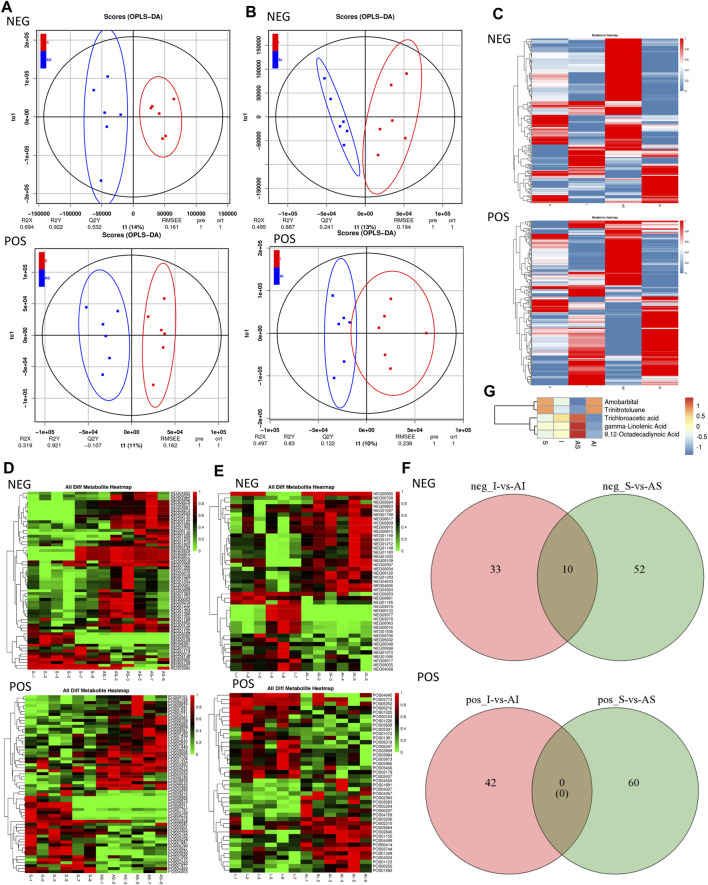
Screening and identification of the differential metabolites in the different groups. The score plot of the metabolic differences between group S and AS **(A)**, and between group I and AI **(B)** by Orthogonal Projections to Latent Structures Discriminant Analysis (OPLS-DA) in both positive and negative ion modes. The heat maps of different metabolites in each sample from the four different groups in both positive and negative ion mode **(C)**. The ionic strengths of both the positive and negative modes of the different metabolites between group S and AS **(D)**, and between group I and AI **(E)** by variable importance in projection (VIP) generated after OPLS-DA. The subsequent Venn diagram in the negative ion mode **(F)**. The mean relative abundance of five known differential metabolites in the different groups **(G)**. S: sham; AS: ARB + sham; I: injury; AI: ARB + injury.

The supervised method, OPLS-DA, was applied to investigate the metabolic differences in different groups and the scores are shown in Figures 5A,B. The results indicated that the plasma samples were clearly separated according to their metabolic profiles of different groups by the score plot of OPLS-DA in both positive and negative ion modes. The segregation was obvious between group S and group AS, and between group I and group AI. The heat maps representing the relative abundance of different metabolites in each sample from the four different groups in both positive and negative ion mode are shown in Figure 5C.

### Screening and Identification of the Differential Metabolites

The VIP generated after OPLS-DA was employed to screen the differential metabolites in samples from different groups. The differential metabolites with VIP values ≥1 were chosen as the differential metabolites responsible for the metabolic profile discrepancy induced by SLV. The ionic strengths from both the positive and negative modes of the different metabolites between different groups are shown in Figures 5D,E. The subsequent Venn diagram (Figure 5F) indicated that in negative ion mode, 10 differential metabolites were found, five of which were unknown, and the mean relative abundance of the five known differential metabolites in different groups is shown. In positive ion mode, none of the metabolites were identified. A total of five metabolites were characterized as potential biomarkers of the pulmonary protective effect of ARB on lung injury in rats (Figure 5G). These candidates were Amobarbital (C1), Trinitrotoluene (C2), Trichloroacetic acid (C3), gamma-Linolenic acid (C4) and 9,12-Octadecadiynoic acid (C5). The mean level of C3, C4 and C5 were significantly down-regulated in group AI compared with that in group I, indicating that after Olm treatment, SLV-induced acute lung injury reduced the production or secretion of these metabolites to reduce the damage caused by SLV. The trend in the mean levels of C1 and C2 between different groups revealed a similar tendency.

### The Correlation Between Differential Metabolites and Lung Microbiota

**FIGURE 6:**
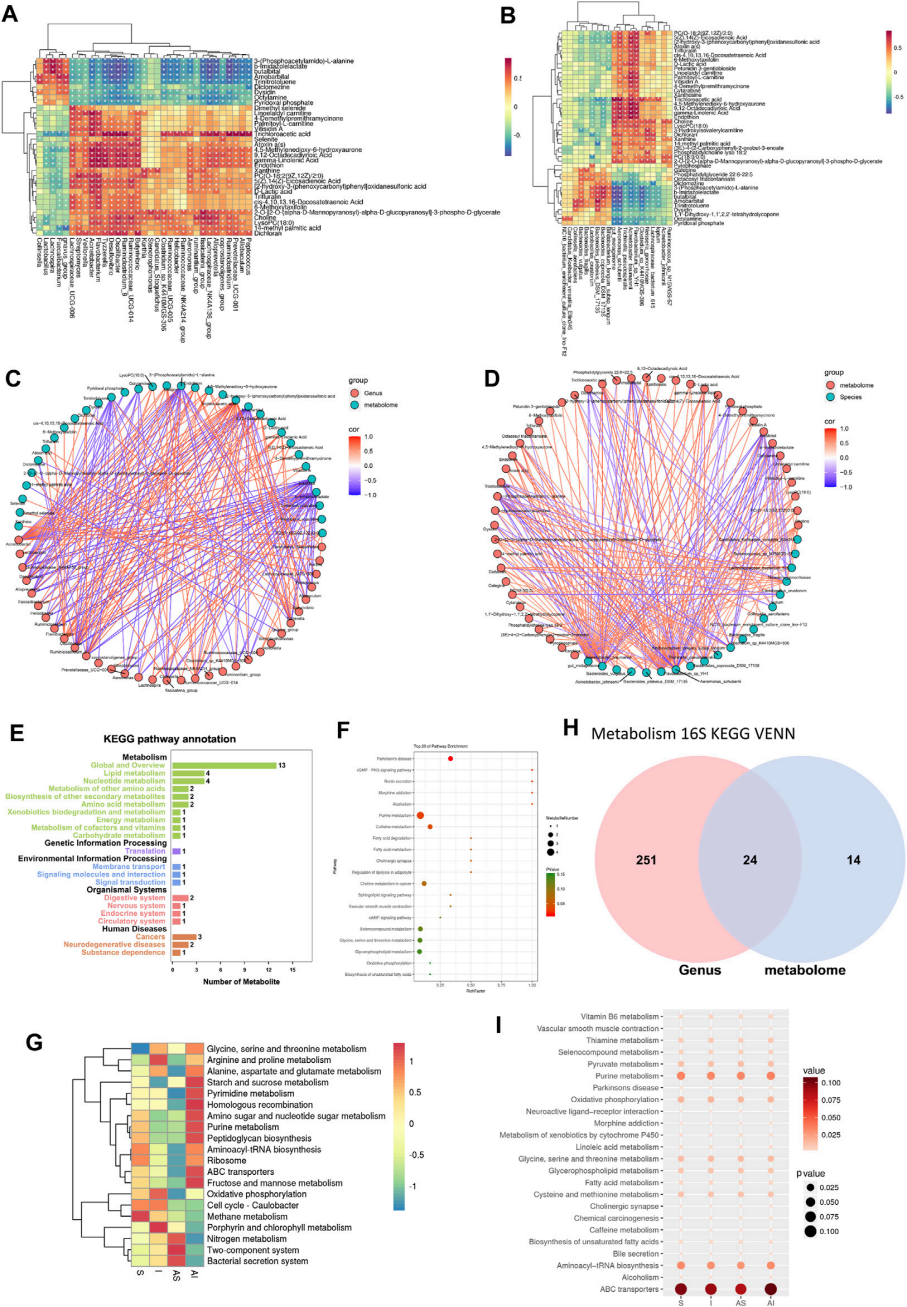
The correlation between differential metabolites and lung microbiota. Correlation heat maps at both genus and species levels are mapped based on O2PLS analysis **(A,B)**. The connections between the lung microbiota and metabolites at both levels are multiple **(C,D)**. KEGG pathway annotation analysis of metabolism **(E)**. Bubble chart from the top 20 pathways enriching with the most metabolites **(F)**. The heat map of the top 20 most relevant pathways according to analysis of the microbial community **(G)**. Venn diagrams of 24 shared pathways **(H)**. Bubble chart from the 24 shared pathways **(I)**. S: sham; AS: ARB + sham; I: injury; AI: ARB + injury; KEGG: Kyoto Encyclopedia of Genes and Genomes; O2PLS: Two-way Orthogonal Partial Least Squares.

Before analyzing the correlation between differential metabolites and lung microbiota, the unknown differential metabolites, and microbiota whose relative abundance was less than 0.1 were excluded. Based on the application of O2PLS analysis, the Pearson correlation coefficient model and canonical correspondence analysis (CCA), correlation heat maps of both genus and species levels were mapped (Figures 6A,B), and only the metabolites and bacteria whose absolute Pearson correlation coefficient value was greater than 0.5 were included. The connections between lung microbiota and metabolites at both levels were multiple, which are shown in the correlation network diagrams (Figures 6C,D).

Additionally, KEGG was applied and significantly enriched pathways were identified to link with differential metabolites and microbiota. A total of 38 pathways were obtained based on the significant differential metabolites, and 20 of them enriched with most metabolites are shown in Figures 6E,F. The 20 most relevant pathways according to analysis of the microbial community are shown in Figure 6G. Finally, 24 shared pathways were identified (Figures 6H,I). Among them, purine metabolism, oxidative phosphorylation, aminoacyl-tRNA biosynthesis and ATP-binding cassette (ABC) transporter were mainly responsible for the lung protective effects of Olm in SLV-induced acute lung injury with microbiota involvement.

**FIGURE 7:**
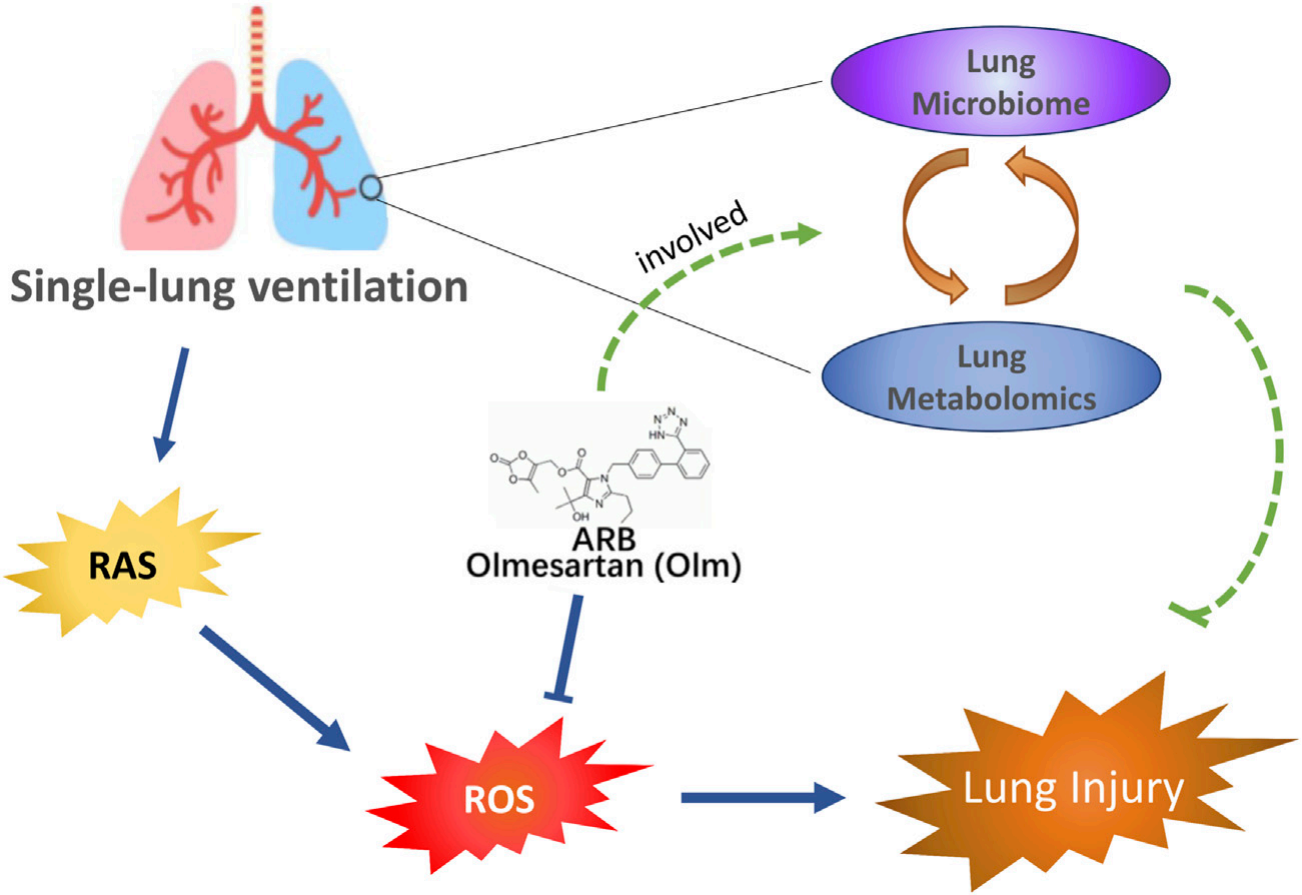
Olm plays a positive role in the prevention of SLV-induced lung injury. In addition to its traditional blockage of the renin-angiotensin II system, Olm may play a positive role in the prevention of SLV-induced lung injury through the pulmonary microbiota and metabolites. SLV: single-lung ventilation; Olm: olmesartan.

In this study, it was demonstrated that Olm may help protect the lung from injury after SLV in rats partly by altering microbial communities in the lung and influencing metabolic pathways and metabolites (Figure 7).

## Discussion

SLV is now widely used due to its ability to improve exposure of operative sites, but it may induce sequential lung injury (Karzai and Schwarzkopf, 2009). Some studies have been sought to explain the occurrence of SLV-induced acute lung injury, including injury caused by inflammation and ROS, thus facilitating lung epithelial and pulmonary vascular permeability, which are further supported by serum markers and histological analysis (Misthos et al., 2005; Ferrari and Andrade, 2015; Gong et al., 2017; Heerdt and Stowe, 2017). To overcome such injury, it is reasonable that more attention should be paid to control inflammation and reduce the generation of ROS.

Over the past few years, the lung microbiome has been reported to be associated with host health and disease (Wu and Segal, 2017). The lung microbiome can induce naive T cells differentiation into Th1 cells, but not Th2 cells, and protect against disease such as neonatal asthma (Wang et al., 2017). In addition, changes in the lung microbiome were related to the generation of Helios-negative Treg cells in the lungs in a PD-L1-dependent manner, which were considered to inhibit an excessive immune response in acute infection (Gollwitzer et al., 2014). It is also reported that the lung microbiome is altered during every lung disease development studied to date, including asthma, cystic fibrosis and pneumonia (Dickson et al., 2014; Teo et al., 2015; Dickson et al., 2016). Dickson et al. (Dickson et al., 2016) established a dysbiosis-inflammation model to explain the relationship between an altered lung microbiome and the host response. Alteration of the lung microbial community, especially the expansion of selected bacteria (e.g., *P. aeruginosa, S. pneumoniae*), could recruit and activate inflammatory cells, while dysregulation of the inflammatory response could alter airway growth conditions and injure prominent microbiota community members. Thus, the exacerbation of disease gradually appeared.

Our 16S rRNA sequencing result and subsequent analysis identified potential candidates, and some of their protective or harmful effects have been reported previously. *Collinsella* was associated with the induction of T helper 17 cells, which modulated the immune system (Rosenbaum and Asquith, 2016). *Clostridiaceae_1_NA* and *Clostridium_sensu_stricto_1* are both members of the family *Clostridiaceae_1,* and *Clostridiaceae_1* was potentially correlated with the severity of allergic airway inflammation (Yang et al., 2019). Thus, our results showed that Olm could play its protective role by decreasing the abundance of such bacteria. *Burkholderiaceae* is pathogenic bacteria and can cause pneumonia-derived sepsis (Lazar Adler et al., 2009). *In vivo* study showed that infection with *Burkholderiaceae* resulted in the release of IL-1β and IL-18, the expression of inflammasome components and the death of lung epithelial cells. It was shown that *Burkholderiaceae* was more enriched in group I compared with group S, but treatment with Olm reduced its abundance in group AI, which was in line with the protective effect of Olm. Researchers have also provided evidence that the signal transduction networks in response to *Mycoplasma* infection might help protect against disease such as lung cancer (Lee et al., 2016; Boyarskikh et al., 2018). Therefore, our study proved that Olm protected SLV-induced lung injury by modulating the abundance of the microbiome partially.

The lung microbiome not only influences the host immune system but also regulates host metabolic homeostasis via microbial metabolites or co-metabolites. In this study, the top four pathways enriched in the lung microbiome’s effects during protection with Olm via metabolites interactions were pyrimidine metabolism, purine metabolism, aminoacyl-tRNA biosynthesis and ABC transporter. These metabolic pathways have previously been reported to be associated with inflammatory regulation (Westerterp et al., 2014; Lee et al., 2018; Linden et al., 2019; Simon et al., 2019). It was challenging to assess the effects of some potential metabolites on lung injury. C1 can block the electron transport chain reversibly to inhibit superoxide generation during ischemia and reperfusion (Chen et al., 2008). C3 is included in anti-inflammation and lipid homeostasis, as a ligand of peroxisome proliferator-activated receptor (PPAR) alpha (Ramdhan et al., 2010). C4 and C5 are involved in the metabolism of arachidonic acid, which can improve lung microvascular permeability, oxygenation and reduce lung inflammation (Hitchcock and Kokolis, 1981; Umar et al., 2011).

Taken together, the significant changes in lung metabolism in rats with acute lung injury might be coupled with oxidative inflammatory response and metabolic disorders. Furthermore, the results of serum markers also proved the potential association between these altered microbiota/metabolites and inflammation. Thus, our study uncovered the paradigm of how Olm attenuated SLV induced lung injury and provided a direct proof that targeting pulmonary microbiota or metabolites is a promising strategy to treat inflammatory and SLV-induced lung injury.

In conclusion, pulmonary microbiota and metabolites may be involved in the positive effect of Olm in alleviating SLV-induced lung injury, in addition to its traditional blockage of the renin-angiotensin II system.

## Data Availability

The original contributions presented in the study are publicly available. This data can be found here: https://www.ncbi.nlm.nih.gov/bioproject/, PRJNA707375.
