# Supplementary material for: Olmesartan Attenuates Single-Lung Ventilation Induced Lung Injury via Regulating Pulmonary Microbiota
Source: Front Pharmacol. 2022 Mar 23;13:822615. doi: 10.3389/fphar.2022.822615 (PMC8984607; doi:10.3389/fphar.2022.822615)
Supplement: Supplementary file 1 [file Table1.DOCX]

Supplemental Table 1. Histological scoring of lung injury

| Parameter | Score per field | | |
| --- | --- | --- | --- |
|  | 0 | 1 | 2 |
| 1. Neutrophils in the alveolar space | None | 1-5个 | ＞5 |
| 1. Neutrophils in the interstitial space | None | 1-5个 | ＞5 |
| 1. Hyaline membranes | None | 1 | ＞1 |
| 1. Proteinaceous debris filling the airspaces | None | 1 | ＞1 |
| 1. Alveolar septal thickening | ＜2X | 2X-4X | ＞4X |

Score= [(20*A) + (14*B) + (7*C) + (7*D) + (2*E)]/100
