# Supplementary figures and images for: Olmesartan Attenuates Single-Lung Ventilation Induced Lung Injury via Regulating Pulmonary Microbiota
Source: Front Pharmacol. 2022 Mar 23;13:822615. doi: 10.3389/fphar.2022.822615 (PMC8984607; doi:10.3389/fphar.2022.822615)

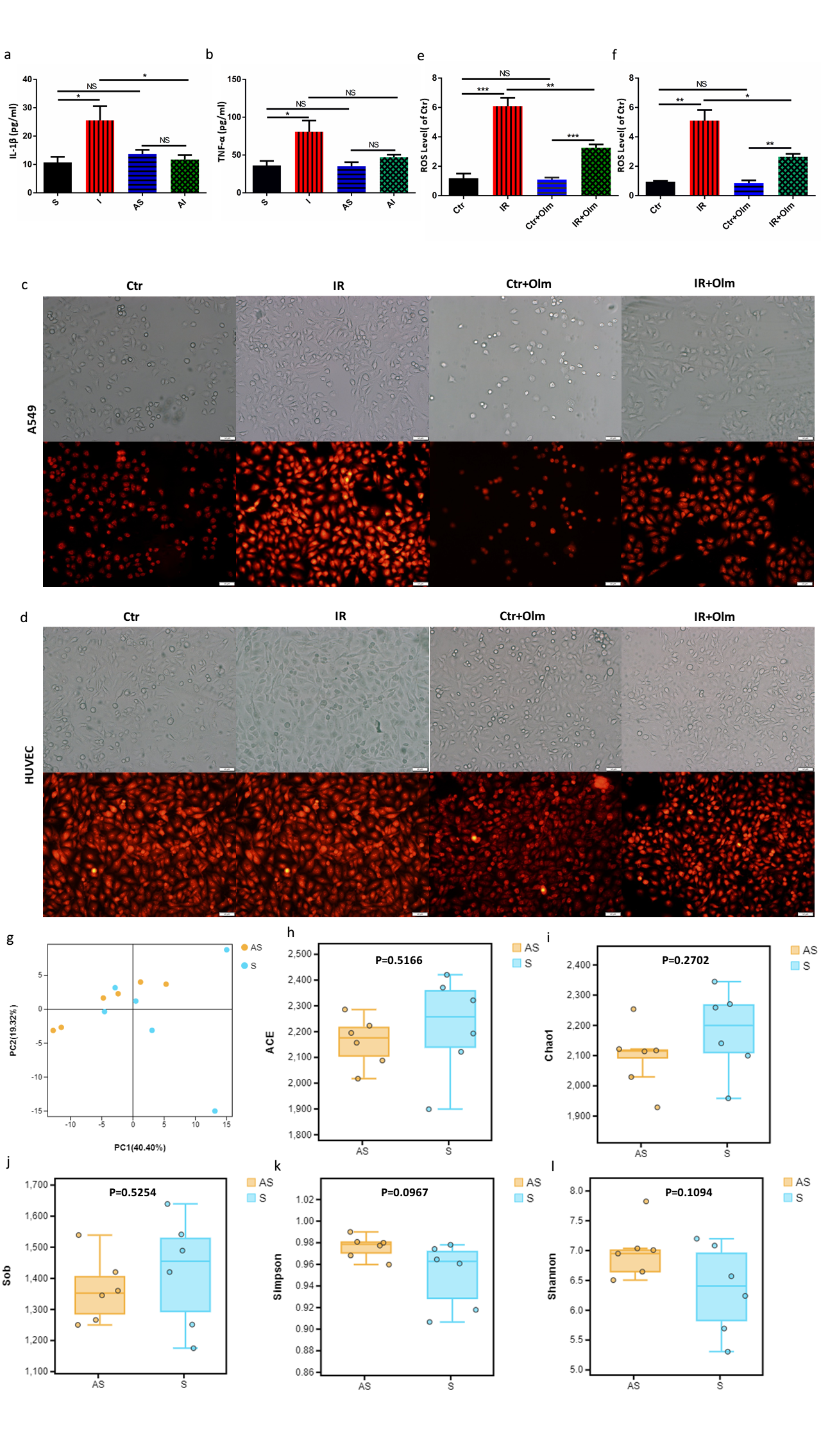

Supplement: Supplementary file 2 [file Image1.TIF]
