# Supplementary material for: Olmesartan Attenuates Single-Lung Ventilation Induced Lung Injury via Regulating Pulmonary Microbiota
Source: Front Pharmacol. 2022 Mar 23;13:822615. doi: 10.3389/fphar.2022.822615 (PMC8984607; doi:10.3389/fphar.2022.822615)
Supplement: Supplementary file 3 [file Table2.DOCX]

Supplemental Table 2 Differential bacteria microbiota between group S and AS at the Species levels

| S vs AS | | | | | | | | | |
| --- | --- | --- | --- | --- | --- | --- | --- | --- | --- |
| Name | | P value | | Name | | P value | Name | P value | |
| group_NA | <0.0001 | | Flavobacterium_NA | | 0.0045 | | Nosocomiicoccus_NA | | 0.0221 |
| Ilumatobacter_NA | <0.0001 | | Fibrobacter_succinogenes_subsp_succinogenes | | 0.0052 | | Candidatus_Koribacter_versatilis_Ellin345 | | 0.0237 |
| Oryzihumus_NA | <0.0001 | | fissicatena_group_NA | | 0.0054 | | Azohydromonas_NA | | 0.0237 |
| hgcI_clade_NA | <0.0001 | | NA | | 0.0054 | | Oscillospira_NA | | 0.0237 |
| Marivita_NA | <0.0001 | | Ruminococcaceae_UCG-014_NA | | 0.0058 | | Oleispira_NA | | 0.0007 |
| Brevifollis_NA | <0.0001 | | Acinetobacter_johnsonii | | 0.0067 | | innocuum_group_NA | | 0.0097 |
| Pseudohongiella_NA | <0.0001 | | bacterium_LWQ8 | | 0.0062 | | torques_group_NA | | 0.0240 |
| Quinella_NA | 0.0001 | | Bacteroides_coprocola_DSM_17136 | | 0.0072 | | Stenotrophomonas_koreensis | | 0.0251 |
| Aeromonas_schubertii | 0.0002 | | Candidatus_Aquiluna_NA | | 0.0067 | | Tyzzerella_NA | | 0.0250 |
| CL500-29_marine_group_NA | 0.0002 | | Candidatus_Planktophila_NA | | 0.0064 | | Comamonas_sp_JL25 | | 0.0268 |
| Fonticella_NA | 0.0002 | | Flavobacterium_sp_YH1 | | 0.0072 | | Collinsella_aerofaciens | | 0.0279 |
| 12up_NA | 0.0003 | | Propionibacterium_NA | | 0.0067 | | Mycobacterium_NA | | 0.0300 |
| Mycoplasma_arginini | 0.0004 | | Synechococcus_NA | | 0.0069 | | Akkermansia_NA | | 0.0312 |
| Mycoplasma_hyorhinis | 0.0004 | | Vibrio_fortis | | 0.0070 | | Candidatus_Stoquefichus_NA | | 0.0320 |
| Kaistia_NA | 0.0004 | | gut_metagenome | | 0.0075 | | Dechlorobacter_NA | | 0.0324 |
| Aliagarivorans_NA | 0.0005 | | OM27_clade_NA | | 0.0080 | | Lactobacillus_NA | | 0.0321 |
| Acinetobacter_NA | 0.0006 | | Veillonella_NA | | 0.0081 | | Ruminiclostridium_9_NA | | 0.0320 |
| Dechloromonas_NA | 0.0006 | | Lachnospira_NA | | 0.0083 | | Streptomyces_NA | | 0.0314 |
| Paucimonas_NA | 0.0007 | | Sphingopyxis_NA | | 0.0093 | | Coprococcus_2_NA | | 0.0329 |
| Succinivibrionaceae_UCG-002_NA | 0.0237 | | Streptococcus_sp_canine_oral_taxon_297 | | 0.0237 | | Bacteroides_plebeius_DSM_17135 | | 0.0335 |
| Aridibacter_NA | 0.0008 | | Empedobacter_NA | | 0.0110 | | Nitrospira_NA | | 0.0338 |
| Rhodanobacter_NA | 0.0009 | | Pseudoxanthomonas_mexicana | | 0.0109 | | eligens_group_NA | | .0411 |
| Planktothricoides_NA | 0.0010 | | coprostanoligenes_group_NA | | 0.0129 | | Shewanella_NA | | 0.0369 |
| Fluviicola_NA | 0.0012 | | CL500-3_NA | | 0.0145 | | Comamonas_aquatica | | 0.0374 |
| Photobacterium_aphoticum | 0.0014 | | Neisseria_gonorrhoeae | | 0.0147 | | Desulfovibrio_NA | | 0.0379 |
| Butyrivibrio_NA | 0.0017 | | ruminantium_group_NA | | 0.0146 | | Ruminococcus_sp_UNKMGS-30 | | 0.0397 |
| Geobacter_NA | 0.0017 | | Bacteroides_fragilis | | 0.0159 | | Bacteroides_vulgatus | | 0.0415 |
| NC10_bacterium_enrichment_culture_clone_Ino-F12 | 0.0017 | | Bifidobacterium_longum_subsp_longum | | 0.0183 | | Lachnospiraceae_ND3007_group_NA | | 0.03660 |
| Anaeromyxobacter_NA | 0.0021 | | Chroococcidiopsis_NA | | 0.0171 | | Ruminiclostridium_NA | | 0.0410 |
| Clostridium_sensu_stricto_10_NA | 0.0021 | | Lachnospiraceae_UCG-004_NA | | 0.0173 | | Anaerovibrio_NA | | 0.0422 |
| Lachnospiraceae_NK4A136_group_NA | 0.0021 | | Marmoricola_NA | | 0.0179 | | Escherichia-Shigella_NA | | 0.0431 |
| Acinetobacter_baumannii | 0.0023 | | Family_XIII_UCG-001_NA | | 0.0164 | | Fusobacterium_NA | | 0.0425 |
| Pedomicrobium_NA | 0.0024 | | Lachnospiraceae_UCG-006_NA | | 0.0195 | | Trichinella_pseudospiralis | | 0.0432 |
| Delftia_tsuruhatensis | 0.0035 | | Bradyrhizobium_NA | | 0.0206 | | Faecalibacterium_NA | | 0.0461 |
| Oscillibacter_NA | 0.0039 | | gnavus_group_NA | | 0.0208 | | hallii_group_NA | | 0.0463 |
| Salinicoccus_NA | 0.0041 | | Kurthia_NA | | 0.0206 | | Ruminococcus_flavefaciens | | 0.0455 |
| Tamlana_crocina | 0.0041 | | Subdoligranulum_NA | | 0.0200 | | Lachnospiraceae_bacterium_615 | | 0.0487 |
| Brevundimonas_NA | 0.0043 | | Terrabacter_NA | | 0.0209 | | Staphylococcus_NA | | 0.0474 |
| group_NA | <0.0001 | | Flavobacterium_NA | | 0.0045 | | Nosocomiicoccus_NA | | 0.0221 |
