# Supplementary material for: Olmesartan Attenuates Single-Lung Ventilation Induced Lung Injury via Regulating Pulmonary Microbiota
Source: Front Pharmacol. 2022 Mar 23;13:822615. doi: 10.3389/fphar.2022.822615 (PMC8984607; doi:10.3389/fphar.2022.822615)
Supplement: Supplementary file 4 [file Table5.DOCX]

Supplemental Table 5 Differential bacteria microbiota between group I and AI at the Genus levels

| I vs AI | | | | | | | | | |
| --- | --- | --- | --- | --- | --- | --- | --- | --- | --- |
| Name | P value | | | Name | P value | | Name | P value | |
| Desulfovibrio | | 0.0006 | Singulisphaera | | | 0.0145 | Acinetobacter | | 0.0333 |
| Turicella | | 0.0008 | bacterium_Ellin6543 | | | 0.0160 | Pirellula | | 0.0338 |
| Planctomycetales_bacterium_Ellin7224 | | 0.0011 | Allobaculum | | | 0.0173 | Akkermansia | | 0.0340 |
| Lysobacter | | 0.0030 | Duganella | | | 0.0173 | fissicatena_group | | 0.0343 |
| Paucimonas | | 0.0039 | group | | | 0.0176 | gnavus_group | | 0.0349 |
| Oscillibacter | | 0.0043 | Burkholderiaceae_NA | | | 0.0183 | Acidobacterium | | 0.0359 |
| Bacteroidales_S24-7_group_NA | | 0.0043 | Ruminiclostridium_9 | | | 0.0197 | Peptococcaceae_NA | | 0.0387 |
| Rhodospirillaceae_NA | | 0.0051 | Pelomonas | | | 0.0203 | Desemzia | | 0.0390 |
| Ruminococcaceae_UCG-005 | | 0.0055 | Nocardioides | | | 0.0213 | Thiopseudomonas | | 0.0390 |
| Terrimonas | | 0.0060 | Ruminiclostridium | | | 0.0231 | Altererythrobacter | | 0.0400 |
| Pseudoxanthomonas | | 0.0065 | Synechococcus | | | 0.0243 | JG30a-KF-32_NA | | 0.0414 |
| Ruminococcaceae_UCG-003 | | 0.0069 | Ruminococcaceae_NK4A214_group | | | 0.0262 | Collinsella | | 0.0419 |
| Armatimonas | | 0.0072 | Helicobacter | | | 0.0270 | Haliangium | | 0.0426 |
| Prevotellaceae_UCG-001 | | 0.0077 | Peptococcus | | | 0.0273 | Anaeroplasma | | 0.0429 |
| Dechloromonas | | 0.0093 | xylanophilum_group | | | 0.0279 | Rhodococcus | | 0.0444 |
| Candidatus_Planktophila | | 0.0109 | Streptomyces | | | 0.0284 | Rheinheimera | | 0.0457 |
| Ruminiclostridium_5 | | 0.0113 | Cytophagaceae_NA | | | 0.0286 | Fimbriimonas | | 0.0467 |
| Roseiarcus | | 0.0128 | Tyzzerella | | | 0.0292 | Clostridium_sp_K4410MGS-306 | | 0.0481 |
| Lactobacillus | | 0.0130 | Clostridiaceae_1_NA | | | 0.0295 | hgcI_clade | | 0.0493 |
| Hungatella | | 0.0134 | Dechlorobacter | | | 0.0322 | Erysipelatoclostridium | | 0.0495 |
| nodatum_group | | 0.0140 | Rs-E47_termite_group_NA | | | 0.0327 | CL500-3 | | 0.0495 |
| Caproiciproducens | | 0.0142 | OM27_clade | | | 0.0329 |  | |  |
