# Supplementary material for: Olmesartan Attenuates Single-Lung Ventilation Induced Lung Injury via Regulating Pulmonary Microbiota
Source: Front Pharmacol. 2022 Mar 23;13:822615. doi: 10.3389/fphar.2022.822615 (PMC8984607; doi:10.3389/fphar.2022.822615)
Supplement: Supplementary file 5 [file Table3.DOCX]

Supplemental Table 3 Differential bacteria microbiota between group S and AS at the Genus levels

| S vs AS | | | | | | | | | |
| --- | --- | --- | --- | --- | --- | --- | --- | --- | --- |
| Name | | P value | | Name | | P value | Name | P value | |
| group | <0.0001 | | Brevundimonas | | 0.0043 | | Bradyrhizobiaceae_NA | | 0.0208 |
| Ilumatobacter | <0.0001 | | Spongiimonas | | 0.0046 | | Bradyrhizobium | | 0.0216 |
| Oryzihumus | <0.0001 | | Burkholderiaceae_NA | | 0.0050 | | Kurthia | | 0.0216 |
| Marivita | <0.0001 | | TM146_NA | | 0.0053 | | gnavus_group | | 0.0218 |
| Brevifollis | 0.0001 | | Clostridiaceae_1_NA | | 0.0055 | | Terrabacter | | 0.0220 |
| Pseudohongiella | 0.0001 | | fissicatena_group | | 0.0056 | | Sutterella | | 0.0225 |
| Quinella | 0.0001 | | Flavobacterium | | 0.0056 | | Nosocomiicoccus | | 0.0232 |
| Fonticella | 0.0001 | | FukuN57_NA | | 0.0059 | | Neisseria | | 0.0235 |
| CL500-29_marine_group | 0.0002 | | bacterium_LWQ8 | | 0.0066 | | Azohydromonas | | 0.0237 |
| Sandaracinaceae_NA | 0.0002 | | Candidatus_Planktophila | | 0.0067 | | Oscillospira | | 0.0237 |
| 12up | 0.0003 | | Propionibacterium | | 0.0069 | | Succinivibrionaceae_UCG-002 | | 0.0237 |
| Cryomorphaceae_NA | 0.0003 | | Candidatus_Aquiluna | | 0.0070 | | torques_group | | 0.0250 |
| MNG7_NA | 0.0003 | | Synechococcus | | 0.0071 | | Tyzzerella | | 0.0259 |
| Acinetobacter | 0.0004 | | Peptococcaceae_NA | | 0.0072 | | alphaI_cluster_NA | | 0.0266 |
| Kaistia | 0.0004 | | Alloprevotella | | 0.0077 | | Lactobacillus | | 0.0288 |
| Aliagarivorans | 0.0005 | | Lachnospira | | 0.0081 | | Bacteroidales_S24-7_group_NA | | 0.0295 |
| Dechloromonas | 0.0006 | | OM27_clade | | 0.0081 | | Stenotrophomonas | | 0.0310 |
| Paucimonas | 0.0006 | | Veillonella | | 0.0084 | | Akkermansia | | 0.0324 |
| Oleispira | 0.0007 | | Methylocystaceae_NA | | 0.0085 | | Streptomyces | | 0.0325 |
| Aridibacter | 0.0008 | | Sphingopyxis | | 0.0094 | | Candidatus_Stoquefichus | | 0.0331 |
| Rhodanobacter | 0.0009 | | innocuum_group | | 0.0099 | | Ruminiclostridium_9 | | 0.0331 |
| Planktothricoides | 0.0010 | | Methylococcaceae_NA | | 0.0108 | | Dechlorobacter | | 0.0334 |
| Fluviicola | 0.0012 | | Comamonadaceae_NA | | 0.0110 | | Coprococcus_2 | | 0.0341 |
| Lachnospiraceae_NK4A136_group | 0.0013 | | Lachnospiraceae_NA | | 0.0112 | | Nitrospira | | 0.0350 |
| 1174-901-12_NA | 0.0015 | | Empedobacter | | 0.0112 | | Staphylococcus | | 0.0359 |
| NC10_bacterium_enrichment_culture_clone_Ino-F12 | 0.0017 | | NA | | 0.0128 | | Caulobacteraceae_NA | | 0.0376 |
| Butyrivibrio | 0.0017 | | coprostanoligenes_group | | 0.0128 | | Lachnospiraceae_ND3007_group | | 0.0377 |
| Geobacter | 0.0017 | | hgcI_clade | | 0.0129 | | Desulfovibrio | | 0.0393 |
| I-10_NA | 0.0020 | | CL500-3 | | 0.0145 | | Nocardioidaceae_NA | | 0.0396 |
| Clostridium_sensu_stricto_10 | 0.0021 | | ruminantium_group | | 0.0146 | | Shewanella | | 0.0396 |
| Pedomicrobium | 0.0024 | | JG34-KF-361_NA | | 0.0163 | | Aeromonas | | 0.0397 |
| Ruminococcaceae_UCG-014 | 0.0024 | | Family_XIII_UCG-001 | | 0.0168 | | eligens_group | | 0.0418 |
| OPB56_NA | 0.0030 | | Chroococcidiopsis | | 0.0175 | | Anaerovibrio | | 0.0426 |
| Delftia | 0.0035 | | Lachnospiraceae_UCG-004 | | 0.0178 | | Collinsella | | 0.0461 |
| Oscillibacter | 0.0039 | | Marmoricola | | 0.0185 | | hallii_group | | 0.0469 |
| B79_NA | 0.0039 | | Lachnospiraceae_UCG-006 | | 0.0201 | | Faecalibacterium | | 0.0470 |
| Salinicoccus | 0.0041 | | Subdoligranulum | | 0.0206 | | Sporichthyaceae_NA | | 0.0491 |
