# Supplementary material for: Olmesartan Attenuates Single-Lung Ventilation Induced Lung Injury via Regulating Pulmonary Microbiota
Source: Front Pharmacol. 2022 Mar 23;13:822615. doi: 10.3389/fphar.2022.822615 (PMC8984607; doi:10.3389/fphar.2022.822615)
Supplement: Supplementary file 6 [file Table4.DOCX]

Supplemental Table 4 Differential bacteria microbiota between group I and AI at the Species levels

| I vs AI | | | | | | | | |
| --- | --- | --- | --- | --- | --- | --- | --- | --- |
| Name | | P value | | Name | | P value | Name | P value |
| Desulfovibrio_NA | 0.0006 | | Allobaculum_NA | | 0.0170 | | Pseudomonas_sp_FS29 | 0.0354 |
| Turicella_NA | 0.0009 | | Duganella_NA | | 0.0173 | | Rheinheimera_NA | 0.0363 |
| Planctomycetales_bacterium_Ellin7224 | 0.0011 | | Rheinheimera_aquimaris | | 0.0173 | | Acinetobacter_johnsonii | 0.0365 |
| Lysobacter_NA | 0.0026 | | group_NA | | 0.0173 | | Trichinella_pseudospiralis | 0.0378 |
| Paucimonas_NA | 0.0035 | | Ruminiclostridium_9_NA | | 0.0191 | | Altererythrobacter_NA | 0.0379 |
| Lactobacillus_crustorum | 0.0037 | | Nocardioides_NA | | 0.0198 | | Aquicella_NA | 0.0388 |
| Oscillibacter_NA | 0.0038 | | Pelomonas_NA | | 0.0198 | | Desemzia_NA | 0.0390 |
| Ruminococcaceae_UCG-005_NA | 0.0049 | | Synechococcus_NA | | 0.0237 | | Lactobacillus_agilis | 0.0390 |
| Lactobacillus_NA | 0.0052 | | Ruminiclostridium_NA | | 0.0240 | | Pseudomonas_pertucinogena | 0.0390 |
| Terrimonas_NA | 0.0053 | | Ruminococcaceae_NK4A214_group_NA | | 0.0251 | | Thiopseudomonas_NA | 0.0390 |
| Pseudoxanthomonas_NA | 0.0057 | | Prevotellaceae_NK3B31_group_NA | | 0.0257 | | Flavobacterium_sp_YH1 | 0.0395 |
| Ruminococcaceae_UCG-003_NA | 0.0060 | | Helicobacter_NA | | 0.0258 | | Haliangium_NA | 0.0397 |
| Armatimonas_NA | 0.0063 | | Peptococcus_NA | | 0.0261 | | Anaeroplasma_NA | 0.0404 |
| Prevotellaceae_UCG-001_NA | 0.0069 | | xylanophilum_group_NA | | 0.0267 | | Pirellula_NA | 0.0409 |
| Dechloromonas_NA | 0.0086 | | Streptomyces_NA | | 0.0271 | | Streptococcus_NA | 0.0415 |
| Candidatus_Planktophila_NA | 0.0103 | | Mycoplasma_hyorhinis | | 0.0275 | | Acinetobacter_NA | 0.0425 |
| Ruminiclostridium_5_NA | 0.0107 | | Tyzzerella_NA | | 0.0279 | | Acinetobacter_baumannii | 0.0426 |
| Roseiarcus_NA | 0.0124 | | Dechlorobacter_NA | | 0.0303 | | Photobacterium_aphoticum | 0.0438 |
| Hungatella_NA | 0.0129 | | OM27_clade_NA | | 0.0309 | | Fimbriimonas_NA | 0.0445 |
| leptum | 0.0136 | | Collinsella_aerofaciens | | 0.0315 | | Mycobacterium_fortuitum_subsp_fortuitum | 0.0452 |
| nodatum_group_NA | 0.0136 | | Akkermansia_NA | | 0.0318 | | Clostridium_sp_K4410MGS-306 | 0.0459 |
| Caproiciproducens_NA | 0.0138 | | fissicatena_group_NA | | 0.0321 | | Erysipelatoclostridium_NA | 0.0472 |
| Singulisphaera_NA | 0.0141 | | Ruminococcus_sp_N15MGS-57 | | 0.0321 | | CL500-3_NA | 0.0473 |
| bacterium_Ellin6543 | 0.0155 | | gnavus_group_NA | | 0.0329 | | gut_metagenome | 0.0482 |
| Lachnospiraceae_bacterium_615 | 0.0160 | | Acidobacterium_NA | | 0.0339 | | hgcI_clade_NA | 0.0483 |
